# Microglial subtypes: diversity within the microglial community

**DOI:** 10.15252/embj.2019101997

**Published:** 2019-08-02

**Authors:** Vassilis Stratoulias, Jose Luis Venero, Marie‐Ève Tremblay, Bertrand Joseph

**Affiliations:** ^1^ Toxicology Unit Institute of Environmental Medicine Karolinska Institutet Stockholm Sweden; ^2^ Departamento de Bioquímica y Biología Molecular Facultad de Farmacia Universidad de Sevilla Sevilla Spain; ^3^ Instituto de Biomedicina de Sevilla‐Hospital Universitario Virgen del Rocío/CSIC/Universidad de Sevilla Sevilla Spain; ^4^ Department of Molecular Medicine Université Laval Quebec QC Canada; ^5^ Axe Neurosciences Centre de Recherche du CHU de Québec‐Université Laval Quebec QC Canada

**Keywords:** disease, heterogeneity, homeostasis, microglia, subtypes, Immunology, Neuroscience

## Abstract

Microglia are brain‐resident macrophages forming the first active immune barrier in the central nervous system. They fulfill multiple functions across development and adulthood and under disease conditions. Current understanding revolves around microglia acquiring distinct phenotypes upon exposure to extrinsic cues in their environment. However, emerging evidence suggests that microglia display differences in their functions that are not exclusively driven by their milieu, rather by the unique properties these cells possess. This microglial intrinsic heterogeneity has been largely overlooked, favoring the prevailing view that microglia are a single‐cell type endowed with spectacular plasticity, allowing them to acquire multiple phenotypes and thereby fulfill their numerous functions in health and disease. Here, we review the evidence that microglia might form a community of cells in which each member (or “subtype”) displays intrinsic properties and performs unique functions. Distinctive features and functional implications of several microglial subtypes are considered, across contexts of health and disease. Finally, we suggest that microglial subtype categorization shall be based on function and we propose ways for studying them. Hence, we advocate that plasticity (reaction states) and diversity (subtypes) should both be considered when studying the multitasking microglia.

## Introduction

**Figure 1:**
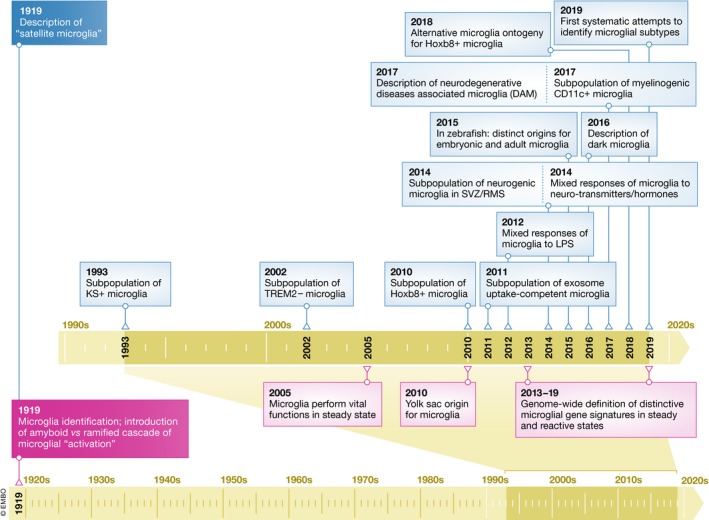
Historical overview of microglial subtype identification Although microglial subtypes have originally been proposed by Rio‐Hortega in the first report of microglia, it was only recently that this idea was revisited.

Microglia were introduced to the scientific literature a century ago (Río‐Hortega, 1919a,1919b,1919c; Fig 1). During normal physiological conditions, microglial cells with a ramified morphology are regularly distributed throughout the central nervous system (CNS; Río‐Hortega, 1919b). Upon pathology, microglia transform their morphology and function, leading to propose a cascade of “reaction” from ramified to hypertrophic and ameboid phenotypes that still orients research today (Flanary *et al*, 2007; Graeber, 2010; Fig 1). With the recent advances in genetic tools allowing for fate mapping (Ginhoux *et al*, 2010), microglia are now considered to be tissue‐resident macrophages of the CNS that arise exclusively from the embryonic yolk sac (Alliot *et al*, 1999; Schulz *et al*, 2012; Kierdorf *et al*, 2013; Perdiguero *et al*, 2015). Microglia colonize the murine CNS from embryonic day (E)9.5 (Tay *et al*, 2017c) and represent a self‐maintaining and long‐lived cell population that persists for months, if not the entire lifespan of the organism (Lawson *et al*, 1992; Ajami *et al*, 2007, 2011; Mildner *et al*, 2007; Askew *et al*, 2017; Füger *et al*, 2017; Réu *et al*, 2017; Tay *et al*, 2017b). Beyond microglia functioning as mediators of injury, inflammation, and neurodegeneration, several roles in the healthy brain have been identified at an exponential rate this past decade (Cartier *et al*, 2014; Tremblay *et al*, 2015; Fig 1).

Microglia exhibit widely differing functions depending on the stage of life, CNS region, and context of health or disease. Differences in microglial number, morphology, and gene expression were also reported between sexes (Schwarz *et al*, 2012; Crain *et al*, 2013; Lenz *et al*, 2013; Pimentel‐Coelho *et al*, 2013; Butovsky *et al*, 2015; Dorfman *et al*, 2017; Hanamsagar *et al*, 2017; Krasemann *et al*, 2017). Adequate microglial functions are crucial for plasticity and behavioral adaptation to the environment (Salter & Stevens, 2017; Tay *et al*, 2017a). Throughout life, microglia contribute to neurogenesis, neuronal circuit shaping, vascular formation and remodeling, and maintenance of homeostasis (Tay *et al*, 2017c). During aging and in diseases, these cells may become reactive or impaired in their surveillance and phagocytosis (Streit, 2002; Koellhoffer *et al*, 2017; Spittau, 2017). Microglial contribution to diseases is associated with compromised physiological roles (e.g., in synaptic maintenance and plasticity; Tay *et al*, 2017a) and processes that are adaptive in the healthy brain, yet leading to cell death and tissue damage in pathological settings (e.g., excitotoxicity, oxidative stress, and inflammation; Weil *et al*, 2008). Microglial reaction can be triggered by any kind of insults or disturbances to the CNS. Persisting microglial reaction, associated often with proliferation, is involved in pathological conditions ranging from neurodevelopmental disorders, traumatic injuries, infectious diseases, tumors, and psychiatric disorders, to neurodegenerative diseases.

**Figure 2:**
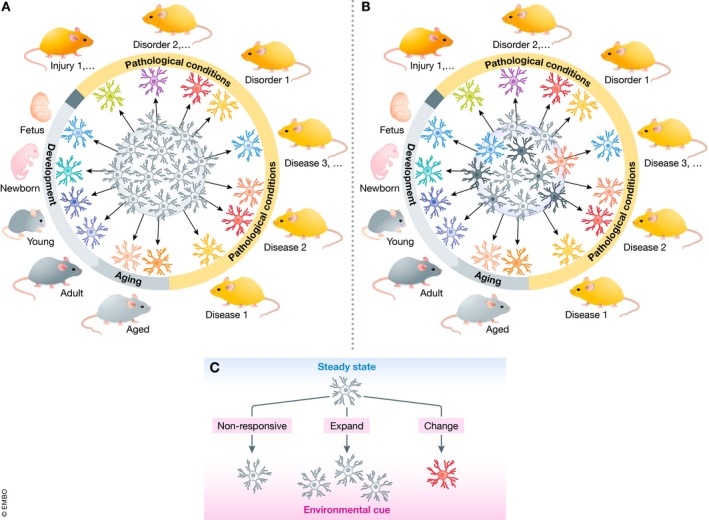
Microglial reaction states (A) Currently, microglia are considered a homogenous cellular population (core of the circle in gray) that is extremely plastic. Depending on the brain homeostasis status at a given developmental stage or resulting from pathology, microglia respond invariably to assume a wide range of phenotypes as described in the literature. (B) In the updated version proposed here, microglia constitute a heterogeneous cell population having intrinsic properties and functional specializations. (C) Upon an environmental cue, each microglial subtype may respond or not to the stimulus, by expanding and/or changing its morphology and gene expression to assume a specific phenotype.

Depending on the stage of the life, CNS region, and stressor or pathological insult at play, the microglial reaction process was shown to proceed differently and to result in sometimes contrasting outcomes (see Fig 2A for a classical schematic representation, depicting a ramified gray microglial cell surrounded by a palette of colorful microglia each representing a distinct reaction state). It is also now recognized that microglia display a wide range of reaction states, a tremendous shift from the M1/M2 classification still used a few years ago (Martinez & Gordon, 2014; Ransohoff, 2016). According to this view, the numerous functions of microglia would be fulfilled through their reaction toward multiple phenotypes, each associated with a distinct molecular signature (Crain *et al*, 2013; Hickman *et al*, 2013; Butovsky *et al*, 2014; Bennett *et al*, 2016; Grabert *et al*, 2016; Flowers *et al*, 2017; Galatro *et al*, 2017; Keren‐Shaul *et al*, 2017; Krasemann *et al*, 2017; Hammond *et al*, 2018; Masuda *et al*, 2019). However, several pieces of evidence also indicate that different pools of microglia might each display distinct intrinsic properties that would be acquired during their maturation or function within the CNS. These subtypes would co‐exist at steady state and undergo further modulation or phenotypic transformation in response to stimuli (Fig 2B). Indeed, beyond the view that microglia are a unique cell type in the CNS that adopts different phenotypes in response to different stimuli, we propose in this review article that microglia might constitute a community of cells in which different members display distinct properties, perform distinct physiological functions, and respond differently to stimuli (Fig 2C). We review the distinctive features of several putative microglial “subtypes”, at structural, ultrastructural, and expression levels, as well as their functional implications across contexts of health and disease. Furthermore, we propose to categorize microglial subtypes based on functions, rather than molecular signatures and markers. Finally, we suggest that microglial subtype candidates should be validated using a methodological workflow that we recommend.

## Microglia: a community fulfilling the vast microglial functions

**Figure 3:**
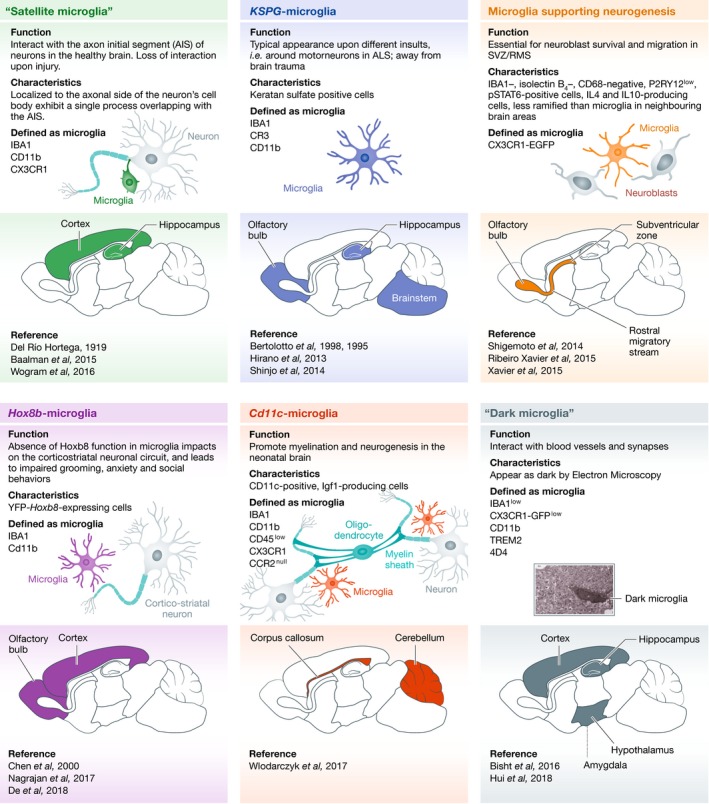
Putative microglial subtypes with unique specializations Emerging data provide support to the existence of putative microglial subtypes endowed with unique genomic, spatial, morphological, and functional specializations. We anticipate that analyzing these subtypes thoroughly, with the methodological workflow proposed in Box 1, and using a similar methodology for newly discovered ones, will result in the identification of a number of different microglial subtypes with unique functional characteristics that could be targeted for disease prevention or treatment.

What defines a cell subtype is subject of intense debate, and it is discussed in Box 1 Accumulating evidence indicates that microglia are not the naïve cell type that invariably responds identically to any possible type of stimuli by assuming a predetermined phenotype. In fact, from a historical perspective, the notion of microglial subtypes had already been proposed in 1919 by Rio‐Hortega in his original description of microglia (Río‐Hortega, 1919b; Fig 1). He noticed that some microglia that he named “satellite” microglia were found in close proximity to neuronal cell bodies. A century later, we propose that the satellite microglia, which are discussed below, might represent one of the playing cards in the deck of microglial subtypes (Fig 3). It is important to acknowledge that others, avant‐garde scientists, have paved the way for the concept of microglial diversity (McCluskey & Lampson, 2000; Olah *et al*, 2011; Hanisch, 2013; Gertig & Hanisch, 2014).

**Box 1: How to define a “cell (sub)type”**

The answer to “how to define a cell subtype?” is probably to be found in the answer to a closely related question, “how to define a cell type?” Traditionally, a cell type is defined based on its host tissue, morphology, lineage, function, and molecular composition. However, the definition of this term remains subject to intense debate (Clevers *et al*, 2017). The advancement of unbiased technologies for single‐cell transcriptome profiling, such as high throughput single‐cell RNAseq and mass cytometry (or improved/related methods), has revealed remarkable heterogeneity among cells which were traditionally considered to be homogeneous. However, whereas this degree of transcriptome and proteome heterogeneity is sufficient for defining cell subtypes, or even cellular states, is also a topic of intense debate (Trapnell, 2015; Okawa *et al*, 2018). While single‐cell RNAseq and mass cytometry allow to define molecularly distinct cell subpopulations, these approaches require to be complemented by the identification of the unique functions associated with these cell populations, in order to define those as cell (sub)types. Worth a notice, it is of importance not to confound cell subtypes with cellular states of reaction. The latter is referring to the different phenotypes and associated functions a cell type may acquire in response to various stimuli. A cell subtype should be defined by shared properties/characteristics within other cells within the cell type. Their unique intrinsic features and selective physiological functions should also be independent from their microenvironment. These two concepts are not mutually exclusive, as a cell subtype in response to a stimulus could react and acquire a new phenotype, i.e., reaction states, thus adding another level of complexity. Microglial subtypes must be defined in steady‐state and unchallenged conditions by their intrinsic propertie(s) which translate into unique physiological function(s).

Typically, the existing literature is the foundation of a research plan, which by definition is biased in respect to studies aimed at identifying a new cell type or subtype. This includes any work with markers, most importantly staining, sorting, and isolation of cells. Reverse genetic approaches can provide a more reliable tool for such studies, but still they have inherit technical limitations such as cell gating in flow cytometry and antibody unspecificity (Luo *et al*, 2013). On the other hand, unbiased technologies such as single‐cell RNAseq, mass cytometry, and electron microscopy are useful tools, but still we should be aware of their limitation in terms of providing a static view of cellular dynamics. They however become useful when combined with two‐photon *in vivo* imaging to provide insights into dynamics. Serendipitous identification is also an approach, but it is sporadic and by definition non‐systematic. All of the above methodologies can contribute to the identification of new microglial subtypes. Considering the various putative subtypes that we have discussed in this review, a need for classifying microglial subtypes is evident. Deciphering whether their variations are instructed by the microenvironment or whether they result from intrinsic properties is of prime importance, using the following methodological workflow: Fate‐mapping strategies allowing to visualize selectively different microglial subsets, for instance using non‐invasive chronic two‐photon *in vivo* imaging—could be performed longitudinally across development, adulthood, and aging, under steady‐state as well as disease conditions—to determine the identity of putative microglial subtypes as microglial subsets or phenotypes. Microglia could be considered subtypes if their defining properties remain when these cells are examined longitudinally, under steady‐state or disease conditions. They would however be considered phenotypes if instead they can transform one into another, notably in response to stimuli. The molecular determinants and physiological roles of the distinct subsets could then be studied using a combination of gene and protein expression analyses, as well as morphology, ultrastructure, and dynamic investigations.

### Microglial regional heterogeneity at steady state

Although microglia are ubiquitously scattered throughout the CNS, their distribution varies across regions, also between the white matter and gray matter (Lawson *et al*, 1990). Microglial morphology differs with the presence of neuronal cell bodies, dendrites and axons, myelinated axons, and blood vessels. Furthermore, microglia exhibit regional differences in self‐renewal and turnover rates under normal physiological conditions and upon stimuli, such as lipopolysaccharide (LPS) challenge (Lawson *et al*, 1992; Ajami *et al*, 2007, 2011; Mildner *et al*, 2007; Askew *et al*, 2017; Füger *et al*, 2017; Réu *et al*, 2017; Tay *et al*, 2017b; Furube *et al*, 2018). The regional microenvironment has been shown to tightly determine microglial identity at the transcriptional level, in both mouse and human (Gosselin *et al*, 2014, 2017). Direct evidence for microglial regional variability notably comes from studies in which microglia were isolated from wild‐type, unchallenged adult mice, according to brain area, and their transcriptome was determined based on panels of pre‐selected microglial markers. In one study, the expression of CD11b, CD40, CD45, CD80, CD86, F4/80, TREM2b, CX3CR1, and CCR9 was compared among microglia isolated from different CNS regions of young adult mice (de Haas *et al*, 2008). Although all of these markers were expressed across the CNS, their protein expression varied significantly between areas. In a similar study performed in adult rats, the expression levels of known microglial markers also showed region‐specific profiles (Doorn *et al*, 2015). Similar studies performed in mice that compared microglia isolated from different brain areas additionally showed regional heterogeneity in expression pattern throughout the lifespan (Butovsky *et al*, 2014; Grabert *et al*, 2016; De Biase *et al*, 2017; Masuda *et al*, 2019). Additionally, in an unbiased single‐cell RNA sequencing (RNAseq) study, in which cerebral tissue and hippocampal tissue from unchallenged young adult mice were analyzed, 47 molecularly distinct cell subtypes were identified, including two belonging to the microglia (Zeisel *et al*, 2015).

These findings raise the intriguing possibility that regional differences in terms of neuronal survival, activity, growth factor release, metabolism, as well as synaptic plasticity, myelination, vascular remodeling, blood–brain barrier properties, may require distinct microglial functions, thus driving the differentiation of distinct microglial subtypes during development or function within the CNS. These microglial subtypes could be a major contributing factor to the microglial regional heterogeneity. Recently, cerebellar microglia were shown to display a unique clearance ability, defined by their expression of numerous genes supporting the engulfment and catabolism of cells or cellular debris (Ayata *et al*, 2018). This cerebellar microglial “type” is reminiscent of developing microglia and disease‐associated microglia (DAM) that will be discussed below. By contrast, microglia from the striatum display a homeostatic surveillance phenotype. This microglial differentiation in response to regional differences in the environment was shown to be driven by epigenetic mechanisms (Ayata *et al*, 2018). In particular, the suppression of clearance genes in striatal microglia is mediated by PRC2, which catalyzes the repressive chromatin modification histone H3 lysine 27 trimethylation (H3K27me3). The ablation of PRC2 in microglia also results in the emergence of clearance microglia even in the absence of dying neurons, among both the striatum and cerebral cortex. These aberrant clearance microglia induce impaired motor responses, decreased learning and memory, together with the development of anxiety and seizures in mice (Ayata *et al*, 2018). A recent study that characterized the diversity of CNS‐associated macrophages (CAM) also identified three different subsets of CAM that expressed high levels of Mrc1, Ms4at, Pf4, Stab1, Cbr2, CD163, and Fcrls, and were associated with different CNS compartments: the leptomeninges, choroid plexus, and perivascular space (Jordão *et al*, 2019). Consequently, some of the regional microglial diversity described using these markers could also be partly accounted for by CAM diversity.

### Microglial subtypes as defined by differential gene expressions

Differential gene expression is an established approach for defining distinct subpopulations of a cell type, for instance the different neuronal subtypes (e.g., GABAergic and glutamatergic) observed in the healthy brain. In various contexts, neighboring microglia were shown to display differences in gene expression at steady state. These observed differences between microglia could arise from local cues, including interactions with different subtypes of neurons (e.g., inhibitory and excitatory) and glial cells (astrocytes, oligodendrocytes, and progenitors), or slight differences in signaling thresholds. Similarly, differences in peripheral macrophage activation by LPS and viruses have been described, where only a subset of the population concomitantly displays a response (Ravasi *et al*, 2002). In addition, microglia may directly communicate with each other, which suggests that the recruitment of a specific microglial cell might lead to an inhibition of the neighboring microglia. Microglia were initially defined as occupying non‐overlapping territories in the healthy brain, but this view is now changing, with improved staining methods showing direct contacts between processes and sometimes cell bodies from neighbor microglial cells (for example, see Milior *et al*, 2016). Furthermore, the possibility that differential marker expression among adjacent microglia results from differences in microglial exposure to previous challenges also has to be considered. For instance, it has been shown using non‐invasive two‐photon *in vivo* imaging that neighbor microglia respond differently to laser injury in the intact, unchallenged brain, leading to their processes converging or not toward the site of injury (Nimmerjahn *et al*, 2005; Paris *et al*, 2018). In addition, microglial cell bodies were recently shown to migrate in the cerebral cortex (Eyo *et al*, 2018) and cerebellum (Stowell *et al*, 2018) of healthy adult mice, which paints another layer of complexity. However, the existence of microglial subtypes, each endowed with intrinsic differences in gene expression, cannot be excluded and we argue that the topic deserves further investigation. Putative microglial subtypes are discussed below:

#### Keratan sulfate proteoglycan (KSPG)‐microglia

**Figure 4:**
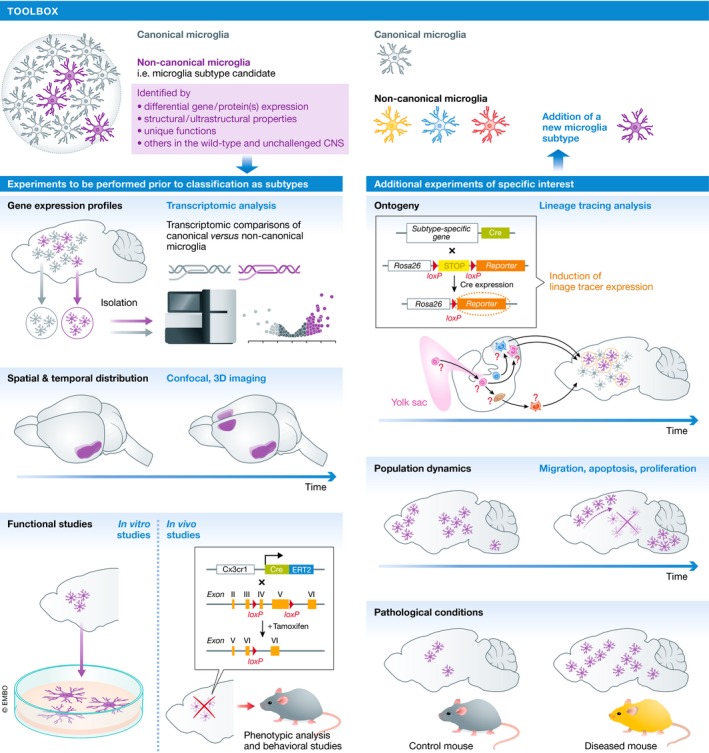
Toolbox.

A quarter of century ago, microglia were shown in the unchallenged adult rat brain to exhibit constitutive heterogeneity in their expression of KSPG (Bertolotto *et al*, 1993), visualized *in situ* using the 5D4 monoclonal antibody (Fig 3). KSPG is located in the extracellular matrix and on the cell surface. They are suggested to contribute to the control of cellular adhesion and axonal growth. In particular, 5D4‐KSPG is expressed by a subpopulation of ramified microglia, contrary to ameboid microglia and peripheral macrophages (Bertolotto *et al*, 1993, 1998). Of note, 5D4‐KSPG expression does not coincide with the expression of GFAP, NG2, or MAP2, which relate to other CNS cells. The expression of 5D4‐KSPG in microglia differs significantly between strains of inbred rats (Jander & Stoll, 1996b). In mammals, a subpopulation of 5D4‐KSPG‐expressing microglia was also reported in the spinal cord and retina (Bertolotto *et al*, 1993, 1998; Jander & Stoll, 1996a; Jones & Tuszynski, 2002; Zhang *et al*, 2005; Foyez *et al*, 2015). The 5D4‐KSPG‐microglia exhibit a preferential regional distribution in the CNS. Indeed, whereas these cells are found in large numbers among the hippocampus, brainstem, and olfactory bulb (OB), only few of them are detected in the cerebellum and cerebral cortex (Bertolotto *et al*, 1993, 1998). This putative microglial subset is also observed in the neonatal rat brain (Bertolotto *et al*, 1998). It is of importance to mention that 5D4‐KSPG‐microglia were shown to co‐exist with 5D4‐KSPG‐negative microglia in the same CNS regions (Jones & Tuszynski, 2002). Although these studies argue for the presence of two different subtypes, based on KSPG‐reactivity, a systematic approach is required to confirm this possibility (Fig 4).

#### Hox8b‐microglia

These microglial cells have a molecular signature that differentiates them from the canonical population, together with a unique spatial and temporal distribution (see Box 2 for distinct ontogeny of *Hoxb8*‐microglia). Mice carrying the driver *Hoxb8‐Cre* and the reporter *ROSA26‐YFP* alleles were crossed to trace YFP‐*Hoxb8* expression. In the adult brain, the only cells showing YFP signal appeared to be microglia. YFP‐positive microglia were found throughout the brain, especially in the cerebral cortex and OB (Chen *et al*, 2010; De *et al*, 2018; Fig 3). YFP‐positive microglia, which represent 25–40% of the total microglial population in the adult brain, were also shown to co‐exist with YFP‐negative microglia (Chen *et al*, 2010; De *et al*, 2018; Nagarajan *et al*, 2018). Transcriptomic analyses comparing *Hoxb8*‐positive and *Hoxb8‐*negative microglia revealed that they are very similar at steady state, with only 21 genes differing significantly in expression between the two populations (De *et al*, 2018). *Hoxb8*‐microglia express microglial signature genes, such as *Tmem119*,* Sall1*,* Sall3*,* Gpr56,* and *Ms4a7*, and genes associated with hematopoietic ontogeny including *Clel12a*,* Klra2,* and *Lilra5* at similar levels compared with non‐*Hoxb8* canonical microglia (Bennett *et al*, 2018; De *et al*, 2018). Of note, neither of the two putative microglial subtypes was found to expresses *Hoxb8* in the adult brain; instead, the lineage tracer approach revealed that *Hoxb8* is expressed by microglial progenitors prior to CNS infiltration (De *et al*, 2018). Selective inactivation of *Hoxb8* in the hematopoietic system was also sufficient to induce pathological grooming behavior, as observed in constitutive *Hoxb8* mutant mice (Chen *et al*, 2010; Nagarajan *et al*, 2018). The strategy for gene deletion included the use of Tie2 Cre mice that affect all hematopoietic cells and endothelial cells (Chen *et al*, 2010). More cell‐specific deletion of Hoxb8 within microglial cells is a prerequisite to determine their selective involvement in pathological grooming behavior.

**Box 2 (with associated illustration): Revisiting the microglial origin(s)**

An important question arising from the existence of microglial subtypes relates to their possible origin(s). Do microglial subtypes possess intrinsic differences prior to populating the CNS, or do they acquire their unique properties once they have assumed their regional distribution within the CNS parenchyma?

Current literature states convincingly that microglia derive from the first wave of hematopoiesis from the embryonic yolk sac in mouse (Ginhoux *et al*, 2010; Hoeffel *et al*, 2015; Perdiguero *et al*, 2015; Sheng *et al*, 2015; Mass *et al*, 2016), where they follow a stepwise maturation program (Mass *et al*, 2016; Matcovitch‐Natan *et al*, 2016), before populating the embryonic brain at E9.5 (Tay *et al*, 2017c). Based on the above literature, microglial subtypes should differentiate once they have assumed their regional distribution inside the CNS parenchyma (a). This hypothesis could explain microglial differences resulting from regional differences in microenvironments or from differences in local cues among the microenvironment such as microglial interactions with different neuronal subtypes (inhibitory, excitatory) and glial cells (astrocytes, oligodendrocytes and their progenitors), or slight differences in signaling thresholds, leading to the observed differences in adjacent microglia. The alternative hypothesis which is based on microglial cells exhibiting intrinsic differences prior to infiltrating the CNS cannot be excluded at this early stage of investigation, and should be tested (b and c). In support of the later hypothesis, Capecchi *et al* reported that *Hoxb8*‐microglia‐progenitors already exist in the yolk sac at E8.5 (De *et al*, 2018). Subsequently, these cells transit through the aorta‐gonad‐mesonephros and fetal liver, where they expand in number, prior to their entry into the brain at E12.5 (De *et al*, 2018) (c). On the same lines, microglial cells found in CSF1R^−/−^ (Ginhoux *et al*, 2010; Erblich *et al*, 2011) and in *IL2‐Tgfb1;Tgfb1* ^−/−^ (Keren‐Shaul *et al*, 2017) transgenic mice are expected to exhibit intrinsic differences prior to infiltrating the brain parenchyma. Recently, it has been reported that at E14.5 two microglial subpopulations exist, based on *Ms4a7* expression (Hammond *et al*, 2018). It would be of great interest to investigate the ontogeny of these two subpopulations. In zebrafish, two waves of microglial infiltration have been reported (Xu *et al*, 2015; Ferrero *et al*, 2018). Microglia of a yolk‐sac‐equivalent structure origin initially populate the embryonic brain. Subsequently, the microglial population is replenished by adult microglia that derive from a distinct tissue later during zebrafish development (d). This microglial diversity could result from species‐specific differences between zebrafish and mouse. Nevertheless, these studies indicate that evolutionarily multiple microglial origins and maturation programs are a possibility. Recently, it has been reported that at steady state a wave of monocytes infiltrate the mouse brain parenchyma at early postnatal stages; however, these cells were rapidly depleted and did not contribute to the later microglial population (Askew *et al*, 2017).

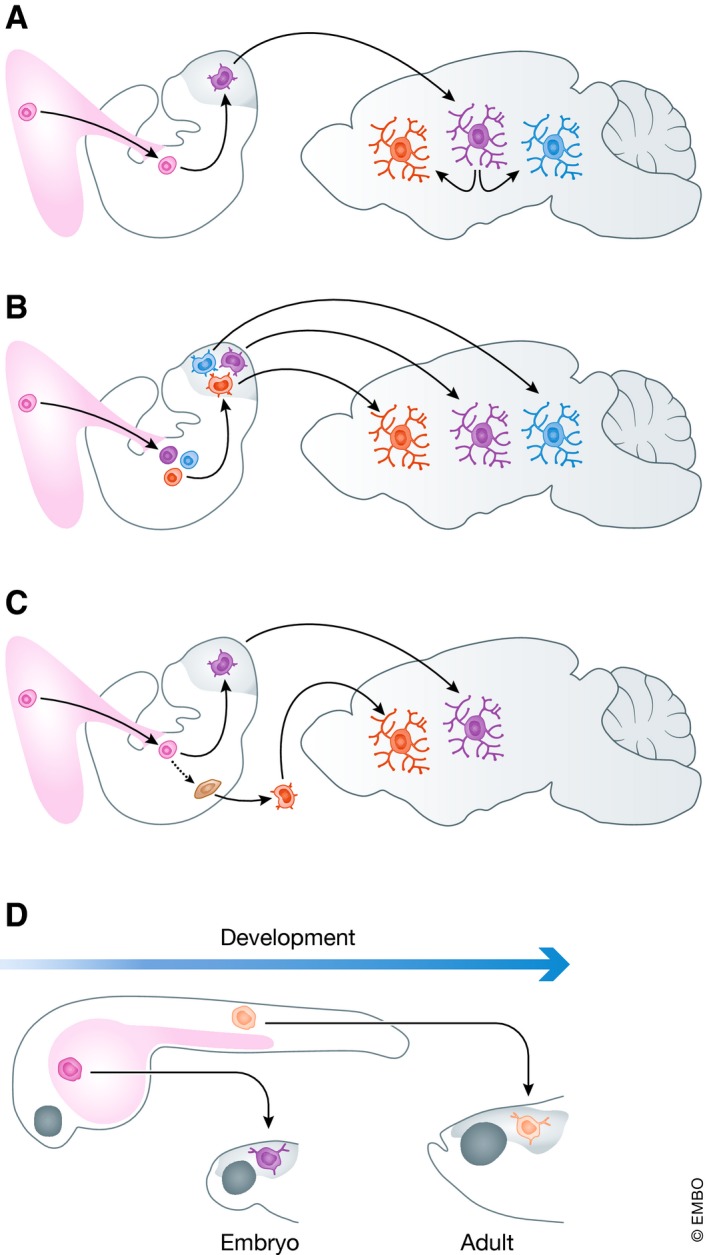

#### CD11c‐microglia

Recently, evidence for an additional microglial subtype expressing the integrin CD11c was uncovered in neonatal mouse brain (Fig 3). *CD11c*‐microglia expand during postnatal development to represent approximately one‐fifth of the total microglial population at postnatal day(P)3‐P5, and then drop to represent less than 3% of the population in juvenile and adult mice (Wlodarczyk *et al*, 2017). Whether this decrease in numbers is due to increased cell death, migration or even trans‐differentiation of this putative microglial subset is yet to be determined. These microglia distribute unevenly across the developing brain, being found predominantly in primary myelinating regions, mainly the corpus callosum and cerebellar white matter. Gene ontology enrichment analysis revealed that *CD11c*‐microglia express genes associated with neurogenic and myelinogenic processes in the neonatal brain. They are also a major source of insulin‐like growth factor 1 (IGF1), while selective depletion of IGF1 in this microglial subtype leads to impaired developmental myelination (Wlodarczyk *et al*, 2017). Thus, *CD11c*‐microglia in the neonatal mouse brain appear to play essential roles in neurogenesis and myelinogenesis during development.

#### TREM2‐microglia

There is additional evidence that differential microglial gene expression patterns define several microglial populations in the healthy brain. For example, not all microglia express the cell surface receptor TREM2, which is known to impact on their survival, proliferation, clustering around amyloid‐beta (Aβ) plaques in Alzheimer's disease (AD) pathology, phagocytosis, and metabolism (Yeh *et al*, 2017). Furthermore, loss‐of‐function variants in the TREM2 protein increase the risk of developing late‐onset AD among other forms of dementia (Colonna & Wang, 2016). However, despite the apparent essential function of TREM2 in microglia, its expression is far from being ubiquitous and homogenous in those cells throughout the brain. In the mouse brain, microglial expression of *TREM2* also varies between and within individual regions (Schmid *et al*, 2002). The numbers of *TREM2*‐expressing cells are highest in the cingulate cortex and lateral entorhinal cortex, and much lower in the hypothalamus and habenula, while some regions, such as the circumventricular organs, completely lack *TREM2* expression. Worth notice, even in brain regions abundant in *TREM2*‐expressing microglia, *TREM2*‐positive and *TREM2*‐negative cells were found in the immediate vicinity to each other (Schmid *et al*, 2002). Regional differences in *TREM2* gene expression, associated with microglial markers, are also observed in the human brain (Forabosco *et al*, 2013). Microarray data generated from 101 healthy control individuals reveal significant regional differences in *TREM2* gene expression between the white matter and cerebellum (Forabosco *et al*, 2013). This regional heterogeneity in *TREM2* expression could point toward a specific subtype.

#### Microglia supporting neurogenesis

The use of *CX3CR1*‐EGFP reporter mice, in which one of the loci of fractalkine receptor *Cx3cr1* is replaced by the gene encoding EGFP, revealed heterogeneity of the microglial cell population within the neurogenic subventricular zone (SVZ), and the adjacent rostral migratory stream (RMS) that terminates into the OB (Ribeiro Xavier *et al*, 2015; Xavier *et al*, 2015; Fig 3). First, it was noticed that microglia located along the SVZ‐RMS‐OB axis are significantly less ramified than microglia from adjacent areas in mice and rats (Shigemoto‐Mogami *et al*, 2014; Ribeiro Xavier *et al*, 2015; Xavier *et al*, 2015). *CX3CR1*‐EGFP expressing microglia were TREM2‐negative, and about half of them also IBA1‐negative in the SVZ and RMS of wild‐type adult mice. By contrast, *CX3CR1*‐EGFP expressing microglia expressed TREM2 in the OB of wild‐type adult mice. Further adding to this diversity, a subset of the latter population additionally expressed CD68 (~35%) and/or isolectin B_4_ (~15%) in the OB (Ribeiro Xavier *et al*, 2015). Significantly increased CD68 expression in microglia was also reported in the OB of adult wild‐type rats (Doorn *et al*, 2015), while a regional distribution of CD68‐positive microglia was reported in the brain of human midterm fetuses (gestational ages of 15–25 weeks; Cho *et al*, 2013). As observed in the SVZ and RMS, IBA1‐negative and IBA1‐positive microglia were also observed in close vicinity to another within the OB. Of note, the antigenic heterogeneity of *CX3CR1*‐EGFP expressing microglia was reported in later development among the SVZ of newborn (P1) and early postnatal (P7) mice, suggesting persistence of these microglial cell populations beyond ontogeny (Xavier *et al*, 2015). In addition, at those developmental ages, CD68 expression was detected in a microglial subset only. It would be of particular interest to determine whether the CD68‐expressing microglia found in the OB of adult mice originate from the ones detected in the SVZ of newborn mice and whether the microglial heterogeneity described above translates into one or more microglial subtypes during adulthood and aging. A recent study has taken advantage of single‐cell RNAseq analysis to uncover regional microglial heterogeneity across different brain regions (Li *et al*, 2019). Contrary to expected, the authors found a remarkable homogeneity of adult homeostatic microglia (enriched in homeostatic genes such as TMEM119 or P2ry12) regardless of the brain tissue of origin. However, a microglial population expressing Clec7a, one of the most upregulated genes in DAM (Keren‐Shaul *et al*, 2017; Krasemann *et al*, 2017), was shown by immunohistochemistry to be restricted to the subgranular zone of the hippocampal dentate gyrus, another neurogenic niche, in addition to the SVZ and RMS. This restrictive pattern of localization suggests the existence of a microglial subtype that exerts a key role in adult neurogenesis. These findings illustrate the usefulness of combining different methodological strategies to uncover novel microglial populations among specific CNS regions.

These selected examples of differential gene expression among small populations of microglia argue for the existence of distinct microglial subtypes. Of importance, in the majority of these cases, the putative microglial subtypes co‐existed with canonical microglia within the same microenvironment. While extrinsic factors such as local cues in the microenvironment, as discussed above, could determine this variability, microglia also appear to have intrinsic differences that warrant further investigation.

### Single‐cell RNAseq data

Recently, there has been an unprecedented influx of data from single‐cell RNAseq studies in support of a significant microglial heterogeneity. Although at this point these pieces of evidence are descriptive and we suggest that they have to be coupled with functional studies to categorize the identified clusters as different microglial subtypes, it is striking to report such a plethora of different potential microglial subtypes. In particular, Stevens and colleagues performed deep single microglial cell RNAseq at different developmental stages and uncovered eight transcriptionally distinct microglial clusters co‐existing in the naïve mouse brain (Hammond *et al*, 2018). Among them, “cluster 2” is characterized by a high expression of *Ms4a* family members, some of which are involved in immune cell functions (Eon Kuek *et al*, 2016), and partially overlaps with brain border macrophage markers (Hammond *et al*, 2018). The number of cells belonging to “cluster 2” decreases drastically during postnatal development. “Cluster 3” is almost exclusively found in the embryonic and early postnatal brain. It is characterized by its unique expression of *Fabp5*, while “cluster 6” highly expresses genes that include *Cd74*,* Ccl24,* and *Arg1* and is enriched in female samples (Hammond *et al*, 2018). “Cluster 4” [named axonal tract‐associated microglia (ATM) by the authors] has a very specific spatiotemporal expression. Microglia belonging to this cluster highly express *Spp1*,* Gpnmb, Igf1*,* CD68,* and *Lgals3*, and they have an ameboid morphology. Furthermore, their number is significantly enriched in the neonatal brain and they are preferentially localized in the white matter, including the corpus callosum, and cerebellum (Hammond *et al*, 2018).

#### Proliferative‐region‐associated microglia (PAM)

Similarly, Barres and colleagues performed deep single microglial cell RNAseq at different developmental stages including late embryonic, early postnatal (P7) and adult ones across different brain regions (Li *et al*, 2019). Three well‐defined microglial clusters irrespective of cell cycle states were identified during postnatal development (Li *et al*, 2019) and initially named P7‐C0, P7‐C1, and P7‐C2. The P7‐C0 and P7‐C1 microglial clusters both expressed homeostatic genes although at lower levels for P7‐C1. This specific cluster expressed many genes recently identified in DAM including *Igf1*,* Spp1*,* Gpnmb*,* CD11c* (also known as *Itgax*), and *Clec7a* (Keren‐Shaul *et al*, 2017; Krasemann *et al*, 2017). Further analysis identified these cells to be predominantly located in the corpus callosum and cerebellar white matter, where they intermingled with Mbp^+^ oligodendrocytes; hence, they were named the proliferative‐region‐associated microglia (PAM). These PAM are highly reminiscent of the *CD11c*‐microglia described by Wlodarczyk *et al* (2017) (Wlodarczyk *et al*, 2017) and the ATM cluster identified by Hammond *et al* (2018). Indeed, PAM populate the white matter at P4, peak at P7, and almost disappear by P14. At the morphological level, PAM are ameboid with thicker primary branches and larger cell bodies, as compared with the typical microglia. They are also highly phagocytic, and contrary to the DAM, they do not depend on either triggering receptor expressed on myeloid cells 2 (TREM2) or ApoE (Keren‐Shaul *et al*, 2017; Krasemann *et al*, 2017). Although it is clear that postnatal PAM may represent a specialized microglial subtype involved in the elimination of oligodendrocytes during myelination, the exact timing and appearance of this microglial population in the white matter during periods of myelination indicates that the environmental needs may be behind the polarization of homeostatic microglia toward PAM.

In a third study, Prinz and colleagues (Masuda *et al*, 2019) analyzed single microglial cells derived from brain areas that were previously shown to exhibit regional transcriptional differences (Grabert *et al*, 2016), namely cortex, cerebellum, and hippocampus, and additionally analyzed microglia from corpus callosum. Microglia from juvenile (3 weeks old) and adult (16 weeks old) animals were grouped into at least four distinct clusters which showed a variable distribution between brain regions and developmental stage (Masuda *et al*, 2019). Importantly, this study also identified multiple different microglial clusters in human tissue, from cortex that bears no signs of CNS pathology (Masuda *et al*, 2019), therefore indicating that microglial heterogeneity is also relevant to human (see also later).

### Microglial subtypes as defined by differential structural/ultrastructural properties

Returning to the satellite microglia described by Río‐Hortega (1919b), this subpopulation is currently identified based on its unique feature of having its soma being associated with neuronal cell bodies (Baalman *et al*, 2015; Wogram *et al*, 2016; Fig 3). Half of the satellite microglia extend a single process that overlaps with the portion of the axon where potentials are initiated (Baalman *et al*, 2015). They have been identified both during development and at adulthood in mice, while they have a preferential association with excitatory neurons (Baalman *et al*, 2015). In addition, this subpopulation was reported in the cerebral cortex of adult rats and adult non‐human primates (Río‐Hortega, 1919b; Baalman *et al*, 2015), hence indicating conservation across species. Whether satellite microglia represent a transitional state of “surveilling” microglia or bear intrinsic differences compared to their neighboring microglia is yet unknown, as no specific “satellite” microglial marker has been identified and transcriptomic analysis of this microglial subpopulation was not performed. Yet, they are preferentially located in the cerebral cortex (~8% of cortical microglia) as well as hippocampus, and to a lesser degree in the thalamus and striatum (Baalman *et al*, 2015; Wogram *et al*, 2016), suggesting that they perform a specialized function. Interestingly, in the adult mouse cerebellum, a dynamically unique population of “satellite” microglia was recently described using non‐invasive two‐photon *in vivo* imaging (Stowell *et al*, 2018). These cells display migration of their soma, which interact with both Purkinje neuronal cell bodies and proximal dendrites. These cerebellar microglia are also less ramified than their cortical counterparts and show reduced surveillance. This finding indicates that satellite microglia are heterogeneous in their dynamics under steady‐state conditions.

In addition to the distinct microglial morphological properties that were revealed by light or fluorescence microscopy, the use of high spatial‐resolution electron microscopy (EM) uncovered alongside to the typical microglia the occurrence of microglia filled by cellular debris, akin to the fat granule or gitter cells initially described by Río‐Hortega (1920), during aging, age‐related loss of sensory function, and Werner syndrome in mice (Tremblay *et al*, 2012; Hui *et al*, 2018b). Recently, EM also allowed to identify an ultrastructurally distinct microglial population, the “dark” microglia, in the adult and aged mouse hippocampus (CA1 region and dentate gyrus), cerebral cortex, amygdala, and hypothalamus. These cells co‐existing with the canonical population display several ultrastructural features of microglia, particularly their size, morphology, long stretches of endoplasmic reticulum, interactions with neurons and synapses, and association with the extracellular space. However, contrary to the typical microglia, they also display markers of oxidative stress like a condensed, electron‐dense cytoplasm and nucleoplasm, giving them a dark appearance in EM, accompanied by Golgi apparatus/endoplasmic reticulum dilation, mitochondrial alteration, and a partial to complete loss of heterochromatin pattern (Bisht *et al*, 2016; Hui *et al*, 2018a). In neurons, alterations to the heterochromatin pattern are linked to cellular stress, aging, and brain disorders such as schizophrenia and AD (Medrano‐Fernández & Barco, 2016).

The physiological significance of these dark microglia has yet to be elucidated, but they appear to be extremely active. The hundreds of dark microglia examined with EM contacted several synaptic elements with their highly ramified and extremely thin processes (Bisht *et al*, 2016). They generally reached for synaptic clefts, while encircling pre‐synaptic axon terminals as well as postsynaptic dendrites and spines. By comparison, typical microglia focally contact instead of encircle synapses (Tremblay *et al*, 2010). Three‐dimensional reconstructions will be required to determine whether the contacted synaptic elements are completely internalized and phagocytosed by the dark microglia, stripped, or encircled and digested extracellularly. Another distinctive feature of these cells is their frequent association with capillaries, with their processes ensheathing the basal lamina. 52% of the dark cells apposed one capillary, and 13% contacted capillaries simultaneously (Bisht *et al*, 2016). This could suggest a possible implication in vascular remodeling or in maintenance of the blood–brain barrier. In fact, recent ultrastructural investigations confirmed a participation of microglial cells, which resemble dark microglia in terms of localization while displaying different ultrastructural features, in the formation of the glia limitans of arteries, capillaries, and veins (Joost *et al*, 2019).

Surprisingly, the dark microglia were also discovered to be abundant during normal brain development, reaching a maximal density approximating 30 cells/mm^2^, in the first two postnatal weeks when synaptic pruning is most pronounced, both in the cerebral cortex and in the hippocampus [(Bisht *et al*)—unpublished observations]. The dark microglia downregulate IBA1, CX3CR1, and P2RY12, but strongly express CD11b, which forms complement receptor 3 involved in microglia‐mediated synaptic pruning (Stevens *et al*, 2007; Schafer *et al*, 2012), in their processes encircling synaptic elements (Bisht *et al*, 2016). They are also negative for MHC class II, CD206, and CD11c. In support of a microglial origin, the dark microglia do not express the marker of inflammatory monocytes 4C12 and show a strong immunoreactivity against the marker of phagocytic microglia 4D4 on their distal processes (Bisht *et al*, 2016). They were observed in CCR2 knockout mice (Bisht *et al*, 2016), where the recruitment of peripheral monocytes to the brain is impaired (Mildner *et al*, 2007).

## Microglial response to stimuli/pathological changes

### Microglial subtypes can respond differently to one stimulus

Different subpopulations, and potentially subtypes, of microglia in the CNS, have been reported to respond differently to an identical stimulus, or to be differently affected by injuries or diseases affecting the CNS. As an illustration, LPS stimulation results in the induction of TNF‐α or inducible nitric oxide (NO) synthase (NOS2) expression only in a subpopulation of microglia *ex vivo* and *in vivo* (Scheffel *et al*, 2012; Kiyofuji *et al*, 2015). Besides, only a subset of cultured primary rat microglia survive to an LPS challenge, an effect that is related to the self‐production of granulocyte‐macrophage colony‐stimulating factor (GM*‐*CSF) and upregulation of its receptor GM‐CSFR (Kamigaki *et al*, 2016). Recently, Furube *et al* (2018) further reported that the administration of LPS in the circumventricular organs affects microglial proliferation locally and in neighboring regions, but not in the cerebral cortex. Thus, microglia respond unequally to an LPS challenge.

*In vivo* experiments in rodents have shown that various peripheral inflammatory stimuli can affect microglial morphology, gene expression, and/or function in the brain (Hoogland *et al*, 2015). Hence, it is reasonable to speculate that microglial heterogeneity could also be influenced by the periphery. Neher and coworkers recently demonstrated that peripherally applied inflammatory stimuli which induce either acute immune training (e.g., single intraperitoneal injection of LPS) or tolerance (e.g., daily injections of low‐dose LPS on 4 consecutive days) in the brain lead to differential epigenetic reprogramming, associated with unique transcriptome profiles of microglia that persist for at least half a year (Wendeln *et al*, 2018). Epigenetic changes, including histone modifications or DNA methylation as well as microRNA expression, are important modifiers of gene expression, and have been involved in cell phenotype regulation and reprogramming and are therefore part of the mechanisms regulating cellular plasticity including microglia (Cheray & Joseph, 2018). These two types of immunological imprinting, i.e.*,* training and tolerance, were shown to impact on the microglial responses to subsequent disease‐associated stimuli, such as β‐amyloidosis or ischemia (Wendeln *et al*, 2018). Worth a note, the authors noted that in that context the global epigenetic and transcriptional changes were relatively modest, and proposed that they might originate from a small number of microglia. Whether this restricted responsive microglial population reflects a limited number of microglial cells that received the required secondary stimulus or the response of microglial subtype(s) showing a different response threshold remains to be explored.

### Microglial subtypes can respond differently to signal withdrawal and depletion attempts

The class III transmembrane receptor tyrosine kinase, CSF1R, is a key regulator of myeloid cell proliferation, survival, and functions (Dai *et al*, 2002; Ginhoux *et al*, 2010; Erblich *et al*, 2011; Elmore *et al*, 2014). CSF1R can be activated by ligation of colony‐stimulating factor 1 (CSF1) and interleukin (IL)‐34. Despite the fact that CSF1 and IL‐34 share affinity for the same receptor, their biological functions do not strictly overlap, as revealed by their distinct spatiotemporal brain expression patterns, the different impact of their gene deletion, and the cellular responses observed upon their binding to CSF1R (Chihara *et al*, 2010; Nandi *et al*, 2012; Wang *et al*, 2012).

In the brain, although CSF1R is primarily expressed by microglia, a subset of hippocampal and cortical neurons were reported to also express this receptor (Wang *et al*, 1999; Luo *et al*, 2013). Homozygous null mutation of the *CSF1R* gene in mice results in a reproducible and robust, yet not complete, loss of myeloid cells defined by their expression of CD45/CD11b/F4/80 markers (Ginhoux *et al*, 2010), and IBA1‐ and F4/80‐double‐positive cells displaying an ameboid morphology (Erblich *et al*, 2011). Similarly, recent *postmortem* human brain examination of a 10‐month‐old individual carrying a homozygous missense mutation in the CSF1R gene revealed a dramatic reduction in IBA1‐positive cells in the brain. Nonetheless, there was a residual IBA1‐positive cell population with an ameboid morphology that was located mainly around blood vessels (Oosterhof *et al*, 2019). Whether this residual population is conclusively microglia requires further investigation.

CSF1R inhibitors (e.g., PLX5622 and AFS98) have been used to deplete microglia (Han *et al*, 2017). These inhibitors have proved very efficient at robustly reducing microglial numbers in adult mice; however, upon treatment cessation microglia rapidly repopulate the brain parenchyma. Although the mechanisms underlying this repopulation are still under investigation, it was recently shown that residual microglia are the only source of repopulation upon PLX5622 treatment (Huang *et al*, 2018b). Similarly, administration of AFS98 at E6.5 and E7.5 (when yolk‐sac erythro‐myeloid progenitors are generated) is sufficient to deplete macrophages in the E10.5 yolk sac, as well as microglia at E10.5 and E14.5. The depletion is transient with microglia repopulating the brain parenchyma partially around E17.5 (Hoeffel *et al*, 2015), and fully during the first postnatal weeks (Squarzoni *et al*, 2014). The above findings argue for a subset of microglia that might not fully depend on CSF1R stimulation for its maintenance. As further illustration, a recent report indicates that upon microglial depletion with PLX5622, the central cornea is repopulated by a subpopulation of microglia which resides in the optic nerve (Huang *et al*, 2018a).

In addition to CSF1R signaling, TGFb1 has been identified as a major differentiation and survival factor for the microglial cell population. In particular, *IL2‐Tgfb1;Tgfb1* ^−/−^ transgenic mice, lacking TGFb1 expression in the CNS, while retaining its expression in T lymphocytes, show a robust loss of microglia, although a microglial subpopulation exhibiting an ameboid morphology, contrary to microglia in age‐matched wild‐type mice, persisted upon this CNS TGFb1 deficiency (Butovsky *et al*, 2014).

### Microglial heterogeneity in disease conditions

The 5D4‐KSPG‐microglial population described above is influenced by pathology. In particular, 5D4‐KSPG expression is increased selectively in a subset of IBA1/CD11b‐positive microglia in the SOD1^G93A^ mouse model and human cases of amyotrophic lateral sclerosis (ALS; Hirano *et al*, 2013; Foyez *et al*, 2015), and in a Wallerian degeneration mouse model of spinal cord injury (Shinjo *et al*, 2014), while it decreases in a Guillain–Barré syndrome rat model of experimental autoimmune neuritis (Matsui *et al*, 2013). Mice deficient in N‐acetylglucosamine 6‐O‐sulfotransferase‐1 (GlcNAc6ST‐1), an enzyme involved in 5D4‐KSPG biosynthesis, also show reduced 5D4‐KSPG expression in the CNS (Zhang *et al*, 2006). Remarkably, *SOD1* ^*G93A*^ *GlcNAc6ST‐1* ^−/−^ mice exhibit a significantly reduced lifespan and accelerated clinical symptoms as compared to SOD1^G93A^ mice (Hirano *et al*, 2013; Foyez *et al*, 2015), suggesting that 5D4‐KSPG‐microglia which expand in SOD1^G93A^ mice may play a beneficial suppressive role in the early pathogenic phases of ALS (Hirano *et al*, 2013).

In addition, the dark microglia which are rarely observed in healthy young adult mice became highly prevalent upon chronic stress, aging, fractalkine signaling deficiency (in CX3CR1 knockout mice), and AD pathology (APP/PS1 model). Signaling between the neuronal fractalkine (CX3CL1) and its unique receptor, CX3CR1, expressed by microglia, is a main mode of neuron–microglia communication in the brain (Paolicelli *et al*, 2014; Arnoux & Audinat, 2015). The number of dark microglia in the hippocampus quadruples in young adult mice after 2 weeks of chronic unpredictable stress as compared to counterparts housed under control conditions. In the CX3CR1 knockouts exposed to stress, the dark microglial population reached approximately half of the typical microglial population. These cells also became more prevalent during normal aging, at 14 months which corresponds to middle age in mice, where their density tripled compared with young adulthood (3 months of age). In age‐matched APP/PS1 littermates, the number of dark microglia corresponded to almost two‐thirds of the typical microglial population (Bisht *et al*, 2016).

In the APP/PS1 mice, the dark microglia associated with Aβ or neuronal dystrophy were TREM2 positive. These dark microglia frequently contained fibrillary Aβ and encircled synaptic elements displaying signs of dystrophy, including an accumulation of autophagic vacuoles. In adult offspring exposed to maternal immune activation and displaying schizophrenia‐like behavior, dark microglial numbers tripled those in 14‐month‐old APP/PS1 mice (Hui *et al*, 2018a). Interestingly, both the schizophrenia‐like behavior and prevalence of dark microglia were exacerbated in males mice (Hui *et al*, 2018a), suggesting sexual dimorphism in these cells. These overall findings suggest that dark microglia could represent a microglial subtype that exerts specialized functions across development, stress‐induced plasticity, and disease. The physiological roles and molecular determinants of these microglial cells warrant further investigation. Fate‐mapping strategies will be required to determine, using non‐invasive longitudinal two‐photon *in vivo* imaging, whether typical microglia can transform into dark microglia under adaptive pressure, or whether the typical and dark microglia represent distinct subsets already from the developmental stages.

Plaque‐associated microglia illustrate how a specific disease‐associated microenvironment can result in the emergence of different microglial phenotypes (or subtypes). In addition to the dark microglia, an age‐dependent increase in *CD11c*‐labeled microglia is associated with Aβ plaques burden in different models of AD, including CVN‐AD (Kan *et al*, 2015) and APP/PS1 (Kamphuis *et al*, 2016) mice. A characteristic feature of these microglial “types” is their high expression levels of *Igf1* (Kan *et al*, 2015; Kamphuis *et al*, 2016), which is reminiscent of the recently defined *CD11c*‐microglia involved in myelination and neurogenesis during development (Wlodarczyk *et al*, 2017), the ATM (Hammond *et al*, 2018), and PAM (Li *et al*, 2019). These observations suggest the existence of distinct microglial subtypes that emerge to exert specialized physiological functions during specific stages of development and that can also be recruited during pathological conditions when there is a need for such functions. Given the role that *CD11c*‐microglia play in myelination in the healthy brain, their presence in demyelinating diseases was expected. In different studies, indeed, the occurrence of a similar *CD11c*‐microglial population was demonstrated in mouse models of demyelination, such as cuprizone (Remington *et al*, 2007; Wlodarczyk *et al*, 2015), experimental autoimmune encephalomyelitis (EAE; Wlodarczyk *et al*, 2014, 2015), and neuromyelitis optica‐like pathology (Wlodarczyk *et al*, 2015). It is important to note that there is no definite proof that disease‐associated *CD11c*‐microglia arise from the population of CD11c‐positive microglia found in the neonatal mouse brain. When technically possible, performing lineage tracing of the initial *CD11c*‐microglial subtype into adulthood, at steady state and upon demyelination, would be very informative.

However, some clues about the origin of these cells may come from recent investigations that identified a microglial signal regulatory protein α (SIRPα)‐dependent signaling pathway that controls the expansion of *CD11c*‐microglia in adult mice (Sato‐Hashimoto *et al*, 2019). Genetic ablation of this membrane protein SIRPα (including selectively in microglia) or of CD47, a physiological ligand of SIRPα, results in the emergence of *CD11c*‐microglia in the white matter of adult mice, thus suggesting that the CD47‐SIRPα signaling pathway normally represses the activation of *CD11c*‐microglia. A small resident *CD11c*‐microglial population was also reported in the normal adult mouse brain (Bulloch *et al*, 2008; Sato‐Hashimoto *et al*, 2019). However, genetic ablation of SIRPα in this small subset of resident *CD11c*‐microglia only, which was obtained by crossing SIRPα‐flox mice and CD11c‐Cre mice, does not promote the expansion of the *CD11c*‐microglial population in adult mice. These data suggest that the increased number of *CD11c*‐microglia, at least in mice deficient for the CD47–SIRPα‐signaling pathway, is not due to the expansion of the resident *CD11c*‐microglial subset, but instead likely results from the acquisition of CD11c expression in microglia that were initially negative for it. Nevertheless, it is important to notice that multiple signaling pathways can control the expansion or emergence of *CD11c‐*microglia. Indeed, in the CNS, CSF1R stimulation by its ligands, i.e., IL‐34 or CSF1, coincides with the expansion of *CD11c‐*microglial population. Treatments with these CSF1R ligands also appear to reduce both demyelination and symptoms in EAE mouse model (Wlodarczyk *et al*, 2018). However, as it was performed for the CD47–SIRPα‐signaling pathways, it remains to be explored whether this CSF1R‐dependent signaling pathway could impact on the expansion of the resident *CD11c‐*microglial population observed at adulthood.

Developing and demyelination‐associated *CD11c*‐microglia share transcriptomic similarity, but they are far from being identical (Kan *et al*, 2015; Kamphuis *et al*, 2016; Keren‐Shaul *et al*, 2017; Krasemann *et al*, 2017; Wlodarczyk *et al*, 2017; Hammond *et al*, 2018; Li *et al*, 2019). Whether these transcriptomic differences define two distinct populations of *CD11c*‐microglia, or differences due to the stage of the lifespan and the particular context of health or injury, for a unique *CD11c*‐microglial subtype, remains unanswered. Furthermore, recently Stevens and colleagues identified and reported the expansion of a *Ccl4*‐microglial subset upon aging and injury (Hammond *et al*, 2018).

Recent studies have exploited massive transcriptomic analyses to uncover microglial reaction states associated with homeostatic (Butovsky *et al*, 2014; Bennett *et al*, 2016), aging (Hickman *et al*, 2013; Grabert *et al*, 2016; Galatro *et al*, 2017), or disease‐associated (Orre *et al*, 2014; Keren‐Shaul *et al*, 2017; Krasemann *et al*, 2017; Mathys *et al*, 2017) conditions. Transcriptional profile studies of microglia isolated from models of aging and different neurodegenerative diseases demonstrated a strikingly similar transcriptional network across these conditions. For instance, two recent studies performing single‐cell RNAseq of microglia (isolated as discussed below) in mouse aging and models of AD, multiple sclerosis, and ALS, defined a distinctive microglial molecular signature of disease defined as (DAM; Keren‐Shaul *et al*, 2017) or “microglia neurodegenerative phenotype” (MgND; Krasemann *et al*, 2017), suggested to be driven by TREM2. This apparent simplicity in the switch from homeostatic microglia to a common disease‐associated phenotype, even in response to varied conditions of altered microenvironment, suggests that the disease‐associated phenotype program may represent a priming response against more specific challenges. Using mass cytometry which allows to investigate changes in microglial proteome, at the single‐cell level, a similar “disease‐associated” microglial phenotype was recently described in aging and AD pathology (APP/PS1 mice; Mrdjen *et al*, 2018). These cells displayed increased expression of CD14, a co‐receptor of the Toll‐like receptor 4, that is activated by Aβ and pro‐inflammatory cytokines such as TNF‐α (Mrdjen *et al*, 2018).

While the disease‐associated program is inherently linked to AD pathology (derived from the use of 5xFAD or APP/PS1 transgenic mice), a recent report by Mathys *et al* (2017) also revealed that this microglial molecular signature may proceed in a heterogeneous fashion. CK‐p25 mice rapidly develop AD‐associated features ranging from neuronal DNA damage and Aβ accumulation (2 weeks after p25 induction) to progressive neuronal and synaptic loss, as well as cognitive impairment (already severe 6 weeks after p25 induction). The short time window allowed to identify microglial populations associated with early‐stage and late‐stage responses. Analysis of the transcriptional dynamics of late response microglia identified small subsets of single cells upregulating specific gene sets, including antiviral and interferon response genes and components of the MHC class II pathway, which displayed extensive variability (Mathys *et al*, 2017). The authors concluded on the existence of two distinct disease‐associated microglial subpopulations. Since the DAM program, described by Keren‐Shaul *et al* (2017), was not associated with changes in antiviral and interferon response genes, different disease‐associated microglial “subtypes” might be behind these different findings. However, CK‐p25 mouse is an AD model of severe neurodegeneration in contrast to the 5xFAD and APP/PS1 models originally used for the identification of DAM (Keren‐Shaul *et al*, 2017; Krasemann *et al*, 2017; Mathys *et al*, 2017). As a model of severe neurodegeneration, the CK‐p25 mouse exhibits important cell‐associated DNA damage, a well‐known inducer of type 1 interferons (Härtlova *et al*, 2015), which may underlie the specific changes in antiviral and interferon response seen by Mathys *et al* (2017).

Further evidence was recently provided by Tay *et al* (2018) performing single‐cell RNAseq analysis of microglia following facial nerve axotomy at different temporal stages of the disease (i.e., peak and recovery) in adult mice. The authors identified a unique TREM2‐independent microglial subtype at the onset of recovery, which upregulated ApoE and Ccl5, in addition to showing ameboid and anucleated morphologies. Additionally, mass cytometry was used to investigate the changes in signaling and cytokine molecular signatures of microglia in three disease contexts: Huntington's disease (HD), EAE, and ALS (Ajami *et al*, 2018). Although defined as relatively homogeneous by common cell surface markers, microglia in fact contain heterogeneous functional subsets based on their cytokine secretion profile. In particular, two distinct cytokine‐secreting microglial subsets that represent the signature of neuroinflammatory conditions, compared with neurodegeneration models, were identified. Although both neuroinflammatory and neurodegenerative models developed double‐positive TNF‐α‐ and GM‐CSF‐producing cells, this subset abundance correlated best with the height of neuroinflammatory conditions in EAE—peak and onset, whereas it was extremely rare or absent in both models of HD and ALS. Conversely, both neurodegenerative conditions showed a unique subset of cells expressing IL‐10, often considered an anti‐inflammatory cytokine, across all three CNS‐resident myeloid populations (i.e., microglia, meningeal macrophages, and perivascular macrophages) before late‐stage disease symptoms (Ajami *et al*, 2018). Using single‐cell RNAseq of CD45‐positive cells, Prinz and colleagues have recently demonstrated an apparent microglia and CAM diversity under different disease conditions including EAE, cuprizone‐induced demyelination, and unilateral facial nerve axotomy (Jordão *et al*, 2019; Masuda *et al*, 2019). Especially relevant was the microglial heterogeneity associated with multiple sclerosis, a view that was confirmed in both experimental mice and humans (Jordão *et al*, 2019; Masuda *et al*, 2019). Thus, three different microglial subsets linked with the EAE‐associated lesion sites were identified in mice and termed daMG2, daMG3, and daMG4 (Jordão *et al*, 2019). A common feature in the three subtypes differing in the expression of specific chemokines, cytokines, and cysteine proteases was their downregulation of the homeostatic markers P2RY12 and TMEM119. In particular, daMG2 showed strong upregulation of CD74, Ctsb, and Apoe, whereas daMG3 showed high levels of Cxcl10, Tnf, and Ccl4 and daMG4 of Ccl5, Ctss, and Itm2b (Jordão *et al*, 2019). Remarkably, the Prinz team performed scRNAseq from brains of patients with multiple sclerosis and analyzed together with healthy human brains (Masuda *et al*, 2019). This study identified three different clusters associated with homeostatic microglia highly expressing TMEM119 and P2RY12. Three microglial clusters were associated with multiple sclerosis (Hu‐C2, Hu‐C3, and Hu‐C8), thus mimicking and validating the data obtained using the mouse models. Common features of the three clusters were increased expression of Apoe and MAFB along with downregulation or even absence of microglial core genes (Jordão *et al*, 2019). These studies support the challenging view that different microglial subtypes may change their phenotypes to generate context‐ and time‐dependent subtypes (as depicted in Fig 2).

A successful isolation of microglia appears critical to identify microglial subtypes. Recent studies aimed at characterizing the molecular signature of microglia at the single‐cell levels under disease conditions using FACS, with different sorting strategies based on either CD45 or CD11c (Keren‐Shaul *et al*, 2017), FCRLS/CD11b or FCRLS/Clec7a (Krasemann *et al*, 2017) or CD11b/CD45 (Mathys *et al*, 2017; Li *et al*, 2019) or CD45, CD11b, and Cx3cr1 (Hammond *et al*, 2018) or CD45, CD11b, Ly6C, and CD206 (Masuda *et al*, 2019). However, positive selection based on “pan‐microglial” markers accepts that such markers exist. Of note, the widely used “pan‐neuronal” marker NEUN and the “pan‐astrocytic” marker GFAP are not expressed in all neurons (Gusel'nikova & Korzhevskiy, 2015) and astrocytes (Walz & Lang, 1998), respectively. Recent studies have successfully isolated microglia by non‐invasive methods such as RiboTag, single nuclei sequencing, and TRAP (Ayata *et al*, 2018; Haimon *et al*, 2018). These methods preclude non‐specific microglial reactivity, associated notably with the induction of immediate early and pro‐inflammatory genes, during FACS‐based whole single‐cell isolation (Ayata *et al*, 2018; Haimon *et al*, 2018; Li *et al*, 2019). The RiboTag and TRAP approaches require the usage of mice that express Cre recombinase under the control of a microglia‐specific gene promoter. Thus, Cx3cr1Cre has been shown to target neurons and tamoxifen‐based Cx3cr1Cre^ER^ mice target not only microglia, but also non‐parenchymal macrophages (Goldmann *et al*, 2016). This limitation is especially relevant under disease conditions associated with blood–brain barrier disruption. However, whole‐cell transcriptomes are usually contaminated by artifacts inherent to tissue dissociation, by phagocytosed material, and by transcripts sequestered away from ribosomes. These limitations of FACS‐based cell strategy are, on the other hand, a strength when performing RiboTag, single nuclei sequencing, or TRAP. Consequently, each technology has its strengths and weaknesses that should be carefully evaluated when facing the experimental design. Each of them along with complementary technology like mass cytometry, successfully employed for identification of signaling and cytokine molecular signatures of microglia under disease conditions (Ajami *et al*, 2018), will help in the better understanding of the microglial diversity.

We conclude this section with the importance of unraveling the functional roles exerted by microglia in diseases and aging, and to develop for this purpose efficient microglial isolation procedures, as required for cutting‐edge single‐cell RNAseq and mass cytometry. At present, there is even uncertainty as to whether the roles of microglia are protective or deleterious, which may depend on subtype, and vary along CNS regions, stages of the lifespan, and contexts of disease. Interestingly, single‐cell RNAseq has been shown, depending on the analysis depth, to detect only abundant transcripts (Peterson *et al*, 2017). Several transcripts and proteins of the DAM did not overlap (Keren‐Shaul *et al*, 2017; Mrdjen *et al*, 2018), while a marked dissociation of mRNA and protein networks was described in microglia exposed to LPS challenge (Boutej *et al*, 2017). It should be noted also, on a positive note, that mass cytometry data could be useful for instructing isolation procedures, through the identification of cell surface markers that can be used (Mrdjen *et al*, 2018). Complementary techniques that should be considered for investigating microglial subtypes, in order to reduce the influence of microglial isolation procedures on their phenotypic transformation, are RiboTag, TRAP, and single nuclei sequencing. Here, changes in translating ribosomes—thus preventing artifacts introduced by tissue dissociation, cargo contamination, and transcripts sequestered from ribosomes (Haimon *et al*, 2018)—and nuclei—which does not require tissue dissociation and can be performed on fresh‐frozen or lightly fixed samples (Habib *et al*, 2017)—are selectively investigated among microglia. Multiple approaches are indeed needed to reveal changes in gene and protein expression, and ultimately function, to solve this exciting mystery.

## Summary and conclusion

The existing literature provides compiling evidence that several microglial subtypes could co‐exist within the CNS. Microglial heterogeneity in terms of regional differences directed by the microenvironment has been proposed before (McCluskey & Lampson, 2000; Olah *et al*, 2011; Hanisch, 2013; Gertig & Hanisch, 2014), but was overshadowed by a more restrictive one‐microglial population view. Here, we propose the existence of microglial subtypes that bear intrinsic differences and we suggest methodological tools that could lead to the identification, classification, and study of putative microglial subtypes. Our recommendations for the investigation of microglial subtypes are presented in Fig 4.

In summary, we should aim at playing with the full deck of microglial subtypes’ cards (Fig 3), which will tremendously help us in our understanding of microglial biological functions and provide us with the tricks required to selectively act on selected subtype(s), and thereby obtain the desired effect from manipulating selected card(s) and not the whole deck. Considering that different microglial cells can exert various functions under normal physiological conditions, depending on their regional distribution, gene, and protein expression, a better understanding of microglial diversity (made of phenotypes and perhaps subtypes also) is required for the future development of better‐targeted therapies for a variety of disease conditions in which these cells were shown to be involved.

## Conflict of interest

The authors declare that they have no conflict of interest.
